# Cancer-associated fibroblasts induce growth and radioresistance of breast cancer cells through paracrine IL-6

**DOI:** 10.1038/s41420-023-01306-3

**Published:** 2023-01-13

**Authors:** Zhaoze Guo, Han Zhang, Yiming Fu, Junjie Kuang, Bei Zhao, LanFang Zhang, Jie Lin, Shuhui Lin, Dehua Wu, Guozhu Xie

**Affiliations:** 1 Department of Radiation Oncology, Nanfang Hospital, Southern Medical University, Guangzhou, Guangdong Province People’s Republic of China; 2 Breast Center, Department of General Surgery, Nanfang Hospital, Southern Medical University, Guangzhou, Guangdong Province People’s Republic of China; 3 Department of Stomatology, Nanfang Hospital, Southern Medical University, Guangzhou, Guangdong Province People’s Republic of China; 4 Department of Radiation Oncology, Affiliated Cancer Hospital & Institute of Guangzhou Medical University, Guangzhou, Guangdong Province People’s Republic of China; 5 Department of Oncology, Huazhong University of Science and Technology Union Shenzhen Hospital (Nanshan Hospital), Shenzhen, Guangdong Province People’s Republic of China

**Keywords:** Radiotherapy, Breast cancer

## Abstract

In breast cancer, the most numerous stromal cells are cancer-associated fibroblasts (CAFs), which are associated with disease progression and chemoresistance. However, few studies have explored the function of CAFs in breast cancer cell radiosensitivity. Here, CAF-derived conditioned media was observed to induce breast cancer cell growth and radioresistance. CAFs secrete interleukin 6 (IL-6) which activates signal transducer and activator of transcription 3 (STAT3) signaling pathway, thus promoting the growth and radioresistance of breast cancer cells. Treatment with an inhibitor of STAT3 or an IL-6 neutralizing antibody blocked the growth and radioresistance induced by CAFs. In in vivo mouse models, tocilizumab (an IL-6 receptor monoclonal antibody) abrogated CAF-induced growth and radioresistance. Moreover, in breast cancer, a poor response to radiotherapy was associated with IL-6 and p-STAT3 expression. These results indicated that IL-6 mediates cross-talk between breast cancer cells and CAFs in the tumor microenvironment. Our results identified the IL-6/STAT3 signaling pathway as an important therapeutic target in breast cancer radiotherapy.

## Introduction

The most common cancer worldwide is breast cancer, the treatment of which frequently includes radiotherapy. However, radioresistance presents a great challenge to improve the efficacy of radiotherapy of breast cancer. For decades, the study of therapy resistance mainly focused on cancer cells, but did not pay attention to the tumor microenvironment (TME). Recently, a number of studies have indicated that therapy resistance is not only determined by cancer cells, but also is regulated by the TME. Cancer cells interact with stromal cells in the TME to determine therapy sensitivity [1, 2]. Therefore, to overcome therapy resistance, it’s necessary to study cancer cell–stromal cell interactions.

The TME of solid cancers contains several types of stroma cells, e.g., inflammatory cells, immune cells, cancer-associated fibroblasts (CAFs), and other cells [3, 4]. The most abundant stromal cells in breast cancer are CAFs, which are involved in the growth, invasion, and chemoresistance of cancer cells [5–7]. For instance, CAFs promote lung metastasis of breast cancer by secreting interleukin (IL)-33 to mediate the immune microenvironment. CAF-derived IL-33 induces type-2 inflammation as well as recruiting eosinophils, neutrophils, and inflammatory monocytes to the metastatic microenvironment, thus promoting breast cancer lung metastasis. Inhibition of IL-33 led to impaired immune cell recruitment as well as type-2 immunity and suppressed breast cancer lung metastasis [8]. Furthermore, CAFs induce acquired chemoresistance of gastric cancer cells by secreting exosomal miR-522, which is promoted by cisplatin and paclitaxel treatment. CAF-derived exosomal miR-522 inhibits ferroptosis of gastric cancer cells by targeting *ALOX15* (encoding arachidonate 15-lipoxygenase) and decreasing lipid-reactive oxygen species (ROS) accumulation, thereby inducing chemoresistance of gastric cancer cells [9]. Moreover, CAFs were implicated in glycolysis metabolism in head and neck squamous cell carcinoma (HNSCC) cells. The glycolysis and growth of HNSCC cells were promoted by CAF-derived hepatocyte growth factor (HGF), which also induced HNSCC cells to secrete fibroblast growth factor (FGF). HNSCC-derived FGF promoted CAF proliferation and HGF secretion, and inhibition of c-Met and the FGF receptor (FGFR) significantly blocked CAF-induced growth of HNSCC cells [10]. However, there have been few studies of CAF function in breast cancer cell radiosensitivity.

IL-6 is a cytokine produced by cancer cells, CAFs, immune cells, and adipose cells in the TME [11]. Studies have indicated that IL-6 promotes the proliferation, metastasis, and chemoresistance of cancer cells, and induces the secretion of pro-inflammatory factors that contribute to an immunosuppressive TME [12, 13]. For instance, co-culture of preadipocytes with breast cancer cells induced their invasion, migration, and proliferation, which was mediated by preadipocyte-secreted IL-6; blocking IL-6 signaling significantly blocked preadipocyte-induced proliferation, migration, and invasion [14]. Moreover, IL-6 mediated the interaction between gastric cancer cells and CAFs, and contributed to chemoresistance of gastric cancer cells; CAF-derived IL-6 induced activation of the signal transducer and activator of transcription 3 (STAT3) pathway reduced chemotherapy-induced apoptosis, thus promoting the chemoresistance of gastric cancer cells; in patients with gastric cancer, a poor response to chemotherapy is associated with the expression level of IL-6 [15]. However, the role of CAF-derived IL-6 in radiosensitivity is incompletely understood.

In this study, we aimed to investigate the function of IL-6 secreted from CAFs on the biological behavior of breast cancer cells. The results showed that CAF-derived IL-6 induces the growth and radioresistance of breast cancer cells both in vitro and in vivo. Breast cancer cell radiosensitivity was enhanced by targeting the IL-6-STAT3 signaling pathway.

## Results

### Patients-derived CAFs induce breast cancer cell radioresistance

**Fig. 1:**
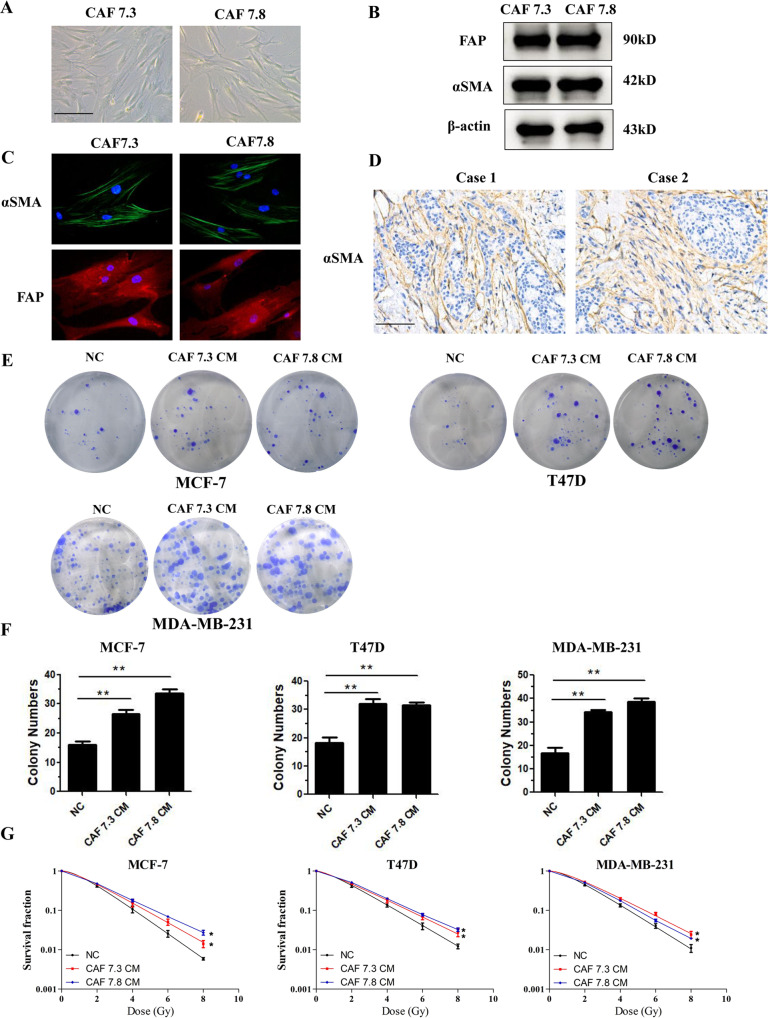
CAFs confer growth and radioresistance of breast cancer cells. **A** Morphological features of primary fibroblasts isolated from fresh breast cancer tissue. Scale bars 50 μm. **B** αSMA and FAP expression in primary fibroblasts was detected using western blotting. All the primary fibroblasts expressed high levels of αSMA and FAP, presenting characteristics of CAFs. **C** Immunofluorescence of αSMA and FAP in primary fibroblasts. **D** IHC labeling of αSMA in breast cancer tissue. Scale bars, 100 μm. **E**, **F** Colony formation assay showing that breast cancer cells treated with CAF conditioned medium formed more colonies than the control cells. **G** Clonogenic survival assay showing that treatment with CAF conditioned medium increased the survival fraction of breast cancer cells. Results are shown as means ± SD, *n* = 3, *P < 0.05, **P < 0.01.

To explore whether CAFs contribute to the radioresitance of breast cancer cells, we isolated primary fibroblasts from fresh cancer tissues from patients who underwent a mastectomy (Table S1). We found that the primary fibroblasts had a myofibroblast-like morphology and expressed the CAF protein markers, smooth muscle actin alpha (αSMA) and fibroblast activation protein alpha (FAP) (Fig. 1A, B and Fig. S1). Furthermore, an immunofluorescence assay showed that the primary fibroblasts were positive for αSMA and FAP expression (Fig. 1C). Moreover, we observed αSMA expression in the stromal cells in the breast cancer tissues from which the primary fibroblasts were isolated (Fig. 1D). These results indicated that the primary fibroblasts had the phenotype of CAFs.

Next, we collected the conditioned medium (CM) from CAFs and used it to culture breast cancer cells. We found that culture with CAF CM increased the colony numbers of breast cancer cells (Fig. 1E, F). Importantly, exposure to CAF CM enhanced the fraction of breast cancer cells that survived after irradiation (Fig. 1G). These results indicated that CAFs promote breast cancer cell growth and radioresistance.

### CAFs enhance the growth and radioresistance of breast cancer cells in vivo

**Fig. 2:**
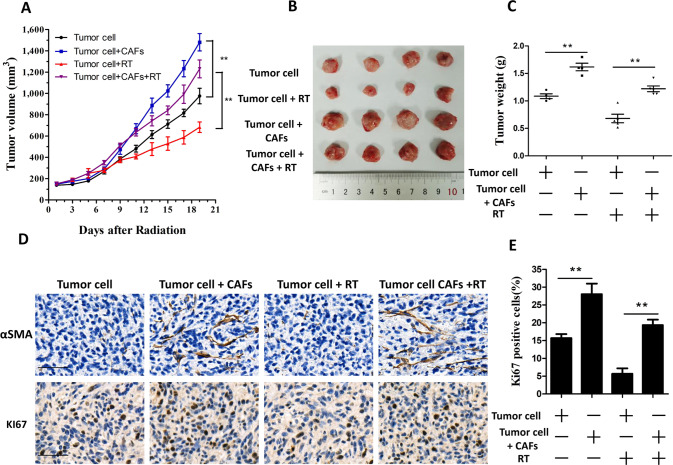
CAFs facilitated the radioresistance and growth of breast cancer cells in vivo. **A** MDA-MB-231 cells (1 × 10^5^) alone or mixed with CAFs (4 × 10^5^) were implanted into the right limb of nude mice. When the tumors grew to 150 mm^3^, the tumors were exposed daily to 2 Gy radiation for five treatments. The tumor volume was monitored every 2 days. Data points indicate the average tumor volume (mm^3^) in each group (*n* = 4); bars, SE. *P < 0.05, **P < 0.01. **B** Image of tumors obtained from the nude mice. **C** The weights of the tumors obtained from the nude mice. Data points indicate the average tumor weight (g) in each group (*n* = 4); bars, SE. *P < 0.05, **P < 0.01. **D**, **E** IHC labeling of αSMA and KI67 in tumors derived from the nude mice. Scale bars, 50 μm. Results are shown as means ± SD, *n* = 3, *P < 0.05, **P < 0.01.

To determine whether breast cancer cell growth and radioresistance is promoted by CAFs in vivo, MDA-MB-231 cells were mixed with CAFs and injected into nude mice following irradiation treatment. We found that the tumors in the CAF co-injection group grew faster than those in the control group. Radiation resulted in significant growth inhibition in the control group, but only slight growth inhibition in the CAF co-injection group (Fig. 2A, B). Moreover, the tumor weights were increased significantly and radiation led to a slight decreased in tumor weight in the CAF co-injection group (Fig. 2C). Immunohistochemistry (IHC) staining showed significantly increased levels of marker of proliferation Ki-67 in the CAF co-injection group compare with that in the control group, (Fig. 2D, E). These results confirmed the CAF-induced growth and radioresistance of breast cancer cells in vivo.

### CAF-derived IL-6 confers breast cancer cell radioresistance

**Fig. 3:**
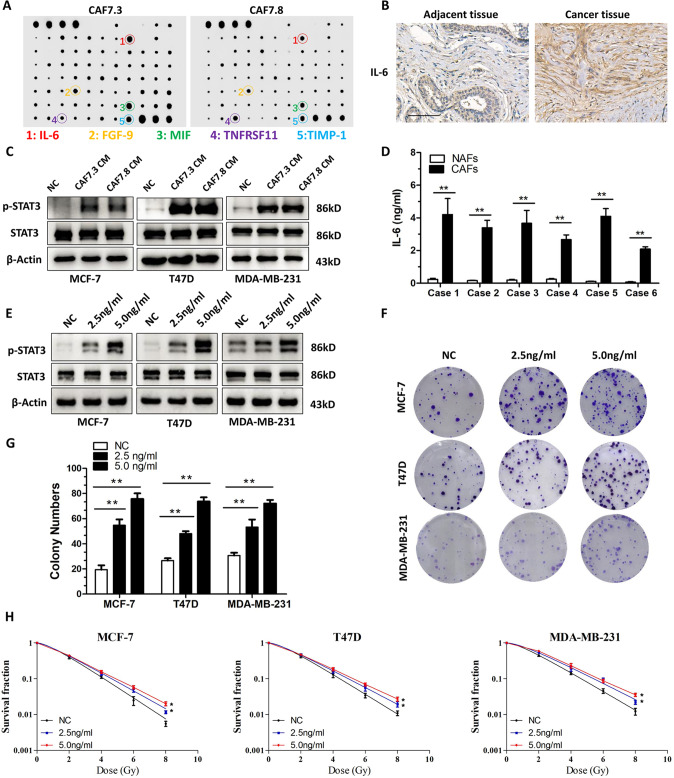
CAFs secreted IL-6 to induce the growth and radioresistance of breast cancer cells. **A** Cytokine antibody array analysis of the growth factors secreted by CAFs. IL-6, FGF-9, MIF, TNFRSF11, and TIMP1 were secreted at high levels from CAFs. **B** The expression of IL-6 was detected via IHC in adjacent normal tissue and breast cancer tumor tissue. Scale bars, 100 μm. **C** Western blotting analysis the p-STAT3 and STAT3 levels in T47D, MCF-7, and MDA-MB-231 cells. The cells were grown in CAF conditioned media for 12 h. **D** The concentration of IL-6 in conditioned media from paired normal tissue-associated fibroblasts (NAFs) and CAFs were analyzed by ELISA. **E** IL-6 was used to treat MDA-MB-231, MCF-7, and T47D cells for 6. The levels of STAT3 and p-STAT3 were analyzed using western blot. **F**, **G** Colony formation assay showing that IL-6 treatment increased the breast cancer cell colony numbers. **H** IL-6 treatment increased the survival fractions of breast cancer cells according to a clonogenic survival assay. The results are displayed as means ± SD, *n* = 3, *P < 0.05, **P < 0.01.

CAFs can secrete a variety of factor to induce the growth and invasion of cancer cells [16, 17]. To investigate which factor mediated CAF-induced growth and radioresistance, we performed a cytokine array assay. The results showed that several factors were highly secreted by CAFs, including IL-6, FGF-9, macrophage migration inhibitory factor (MIF), and TNF receptor superfamily member 11b (TNFRSF11) (Fig. 3A). IL-6 is important inflammatory factor that can activate the STAT3 signaling pathway to induce chemoresistance and radioresistance in a variety of cancer cells [18–20]. Therefore, we hypothesized that IL-6 mediated the CAF-induced radioresistance of breast cancer cells. Consistently, IHC staining showed that compared with that in adjacent normal tissue, the stromal cells in breast cancer tissues expressed high levels of IL-6 (Fig. 3B). Moreover, we found that the level of phosphorylated STAT3 (p-STAT3) increased significantly in breast cancer cells cultured with CAF CM (Fig. 3C and Fig. S2). The concentrations of IL-6 in conditioned media from paired normal tissue-associated fibroblasts (NAFs) and CAFs was detected by ELISA. The results confirmed that IL-6 was secreted highly in CAFs compared with NAFs (Fig. 3D).

To determine whether IL-6 confers radioresistance on breast cancer cells, different concentrations (2.5 ng/ml and 5.0 ng/ml) of recombinant IL-6 was added to culture medium of breast cancer cells. Recombinant IL-6 significantly enhanced the level of p-STAT3 (Fig. 3E) and resulted in increased clone numbers of breast cancer cells in a concentration dependent manner (Fig. 3F, G and Fig. S3). Importantly, the fraction of breast cancer cells that survived after irradiation increased significantly after IL-6 treatment in a concentration dependent manner (Fig. 3H). These findings implied that IL-6 from CAFs induces breast cancer cell growth and radioresistance.

### Inhibition of the IL-6-STAT3 pathway blocked CAF-induced growth and radioresistance of breast cancer cells

**Fig. 4:**
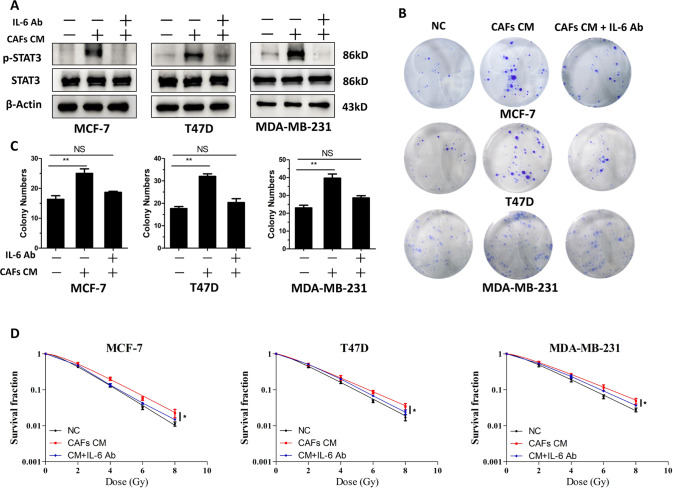
IL-6 neutralizing antibodies blocked the growth and radioresistance induced by CAFs. **A** Western blotting analysis of STAT3 and p-STAT3 levels in T47D, MCF-7, and MDA-MB-231 cells. CAF conditioned medium alone or in combination with IL-6 neutralizing antibody (500 ng/ml) was used to treat the cells. **B**, **C** Colony formation assay showing that treatment with IL-6 neutralizing antibodies blocked the cell growth induced by CAFs. **D** Clonogenic survival assays showing that treatment with IL-6 neutralizing antibodies abrogated the radioresistance induced by CAFs. Results are displayed as means ± SD, *n* = 3, *P < 0.05, **P < 0.01.

To confirm whether IL-6 mediated CAF-induced breast cancer cell growth and radioresistance, CAF CM containing IL-6 neutralizing antibodies were incubated with breast cancer cells. IL-6 neutralizing antibody treatment blocked the CAF CM-induced phosphorylation of STAT3 (Fig. 4A and Fig. S4). Moreover, IL-6 neutralizing antibody treatment blocked the CAF-induced increase in the clone number of breast cancer cells significantly (Fig. 4B, C). Importantly, exposure to IL-6 neutralizing antibodies abolished the radioresistance induce by CAFs (Fig. 4D).

**Fig. 5:**
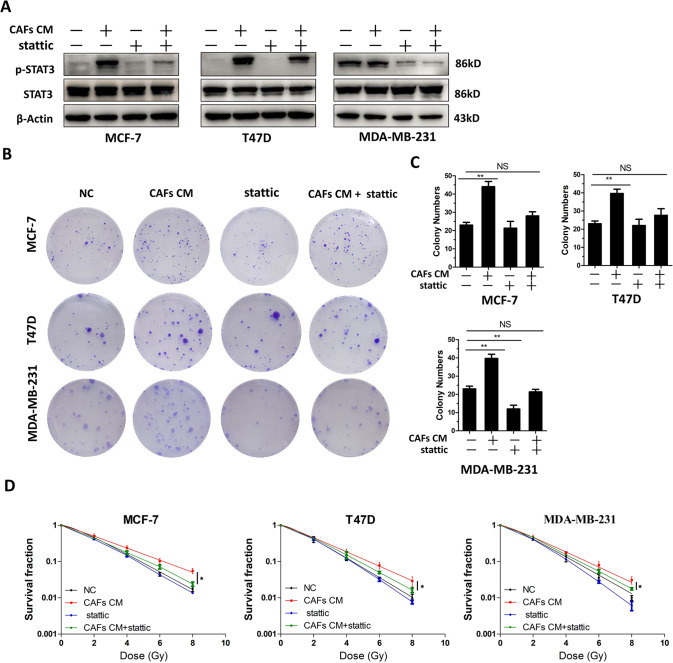
A STAT3 inhibitor abrogated the growth and radioresistance induces by CAFs. **A** Western blot analysis p-STAT3 and STAT3 expression in T47D, MCF-7, and MDA-MB-231 cells. CAF conditioned medium and/or the STAT3 inhibitor Stattic (10 μmol/ml) were used to treat the cells. **B**, **C** Colony formation assay showing that treatment with Stattic blocked the growth induced by CAFs. **D** Clonogenic survival assays showing that treatment with Stattic abrogated the radioresistance induced by CAFs. Results are displayed as means ± SD, *n* = 3, *P < 0.05, **P < 0.01.

We next determined whether the STAT3 inhibitor, Stattic, could block CAF-induced growth and radioresistance. Stattic was add to CAF CM before it was used to culture breast cancer cells. Stattic effectively abolished the upregulation of p-STAT3 induced by CAFs (Fig. 5A and Fig. S5). Furthermore, Stattic treatment abolished the increased in the clone number of breast cancer cells induced by CAFs (Fig. 5B, C). Finally, CAF-induced breast cancer cell radioresistance was reversed by exposure to Stattic (Fig. 5D). These findings indicated CAF-derived IL-6 activates the STAT3 signaling pathway to induce the growth and radioresistance of breast cancer cells.

### Tocilizumab reversed the CAF-induced radioresistance of breast cancer cells in vivo

**Fig. 6:**
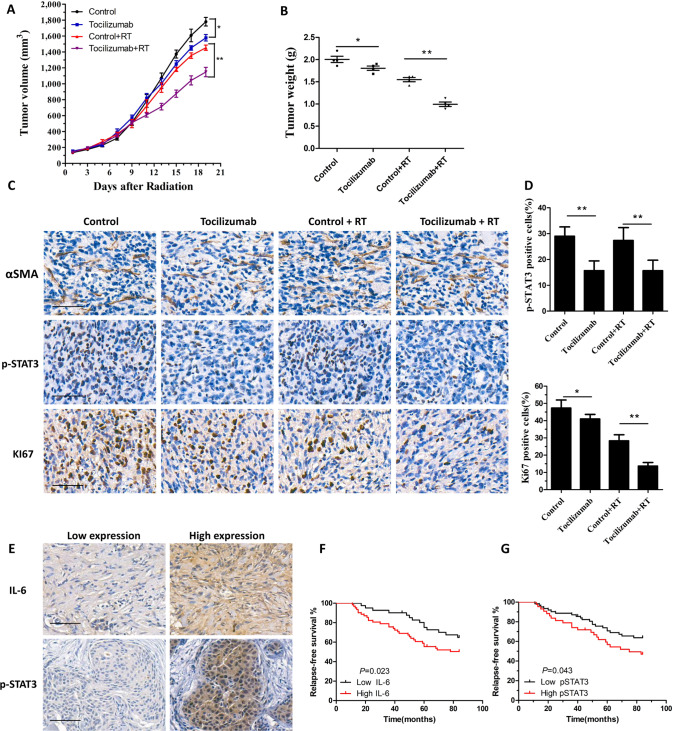
Tocilizumab blocked CAF-induced growth and radioresistance in vitro. **A** MDA-MB-231 cells (1 × 10^5^) and CAFs (4 × 10^5^) were injected subcutaneously into the right limbs of nude mice. When the tumors reach 150 mm^3^, they were exposed daily to 2 Gy radiation for five treatments. Tocilizumab was administered 2 h before irradiation. The tumor volume was monitored every 2 days. **B** The weight of the tumors harvested from nude mice. Data points indicate the average tumor weight (g) of each group (*n* = 4); bars, SE. *P < 0.05, **P < 0.01. **C**, **D** IHC staining of αSMA, p-STAT3, and KI67 in the tumors derived from the nude mice. Scale bars, 50 μm. **E** The levels of IL-6 and p-STAT3 were detected via IHC in breast cancer samples. Scale bars, 100 μm. **F** Kaplan–Meier survival analysis of the relapse-free survival of patients with breast cancer with high tumor levels of IL-6. **G** Kaplan–Meier survival analysis of the relapse-free survival of patients with breast cancer with high tumor levels of p-STAT3. Results are indicated as means ± SD, *n* = 3, *P < 0.05, **P < 0.01.

Tocilizumab is monoclonal antibody targeting IL-6R that has been approved to treat rheumatoid arthritis [21, 22]. To investigate whether tocilizumab can impair CAF-induced breast cancer cell growth and radioresistance in vivo, CAFs and MD-MB-231 cells were mixed and co-injected into nude mice. Tocilizumab was then administrated by intraperitoneal injection. We found that tocilizumab treatment inhibited tumor growth. Importantly, tocilizumab plus radiation treatment led to significantly inhibited tumor growth compare with tocilizumab treatment or radiation alone (Fig. 6A). The weight of the tumors in the tocilizumab plus radiation treatment group were decreased significantly compare with those in the tocilizumab treatment or radiation alone groups (Fig. 6B). IHC staining showed that tocilizumab treatment resulted in significantly decreased p-STAT3 levels in the tumors (Fig. 6C, D). Moreover, the levels of KI67 were reduced in tumors derived from the tocilizumab plus radiation treatment group (Fig. 6C, D). These results indicated that tocilizumab treatment reversed CAF-induced growth and radioresistance of breast cancer cells significantly in vivo.

### IL-6 and p-STAT3 levels were associated with poor response to radiotherapy in patients with breast cancers

IHC was carried out to analyze the levels of IL-6 and p-STAT3 in a cohort of 103 patients with breast cancer. Clinical pathologic factors of the patients were shown in Table S2. All the patients received radiotherapy after surgery. We found that the stromal cells in breast cancer tissues were positive for IL-6, suggesting that IL-6 in breast cancer primarily originates from CAFs (Fig. 6E). Importantly, the IL-6 level correlated significantly with worse disease-free survival of patients with breast cancer (Fig. 6F). Moreover, we revealed that patients with breast cancer with high levels of p-STAT3 had significantly worse disease-free survival compared to those with low levels of p-STAT3 (Fig. 6G). Thus, in patients with breast cancer, a poor response to radiotherapy correlates with IL-6 and p-STAT3 levels.

## Discussion

Radioresistance is still a major obstacle for radiotherapy of breast cancer. Both cancer cells and stromal cells in the TME determine radioresistance. Herein, we found that CAF-secreted IL-6 activated the STAT3 signaling pathway to promote breast cancer cell growth and radioresistance. CAF CM-induced radioresistance was blocked by using an inhibitor of STAT3 or IL-6 neutralizing antibodies. Moreover, in patients with breast cancer, a poor radiotherapy response correlated with high IL-6 and p-STAT3 levels. These findings indicated that in breast cancer cells, IL-6 mediated CAF-induced growth and radioresistance. Our results identified the IL-6-STAT3 signaling pathway as an important therapeutic target for radiotherapy of breast cancer.

We demonstrated that CAFs secreted high levels of IL-6 and IHC staining showed higher levels of IL-6 in stromal cells in breast cancer tissue compared with that in adjacent normal tissue. Previous studies indicated that IL-6 mediates cross-talk between tumor cells and CAFs in the TME [23, 24]. For instance, CAF-secreted IL-6 induces STAT3 pathway activation, thus promoting epithelial-to-mesenchymal transition, growth, invasion, and chemoresistance of cancer cells in esophageal adenocarcinoma [25]. Moreover, colorectal cancer cell growth and progression are promoted by CAF-derived IL-6; a vicious circle of colorectal cancer growth and progression is formed when IL-6 from CAFs induces STAT3 activation, which promotes the production of autocrine IL-6 in CAFs [26]. However, the function of CAF-derived IL-6 in breast cancer cell radiosensitivity is unknown.

In this study, we showed that CAF-derived IL-6 induced STAT3 activation, which promoted breast cancer cell growth and radioresistance. Both STAT3 inhibitor and IL-6 neutralizing antibody could attenuate the growth and radioresistance induced by CAFs. These results indicate that the breast cancer cell growth and radioresistance induced by CAFs is dependent, at least partially, on the IL-6-STAT3 pathway. Additionally, previous studies have shown that IL-6 could attenuated the p53 response to chemotherapy via the JAK/STAT signaling pathway in cancer cells with wild type p53 [27, 28]. P53 expression can be modulated by IL-6 and/or STAT3 by various mechanisms, including influencing TP53 gene transcription and p53 protein degradation [27, 29–31]. Therefore, p53 expression modulated by IL-6/STAT3 axis in cancer cells could be a potential mechanism involved in radioresistance.

Over the last decade, a number studies have reported on the essential role played by the TME in determining therapy resistance and cancer progression; therefore, disrupting the active cross-talk between stromal cells and cancer cells in the TME is considered to be a potential therapeutic strategy to treat cancer [32]. Our study highlighted the critical contribution of IL-6 to the dynamic interaction between CAFs and cancer cells in the TME. Thus, the IL-6-STAT3 signaling pathway has emerged as major therapeutic target for breast cancer radiosensitization.

CAFs are a heterogeneous cell population of diverse cellular origins, and different CAF subpopulations may exhibit different functions [33]. For example, integrin α11 and platelet derived growth factor receptor beta (PDGFRβ)–positive CAFs can active the PDGFRβ-C-Jun N-terminal kinase 1 (JNK) signaling pathway to produce preinvasive matricellular protein tenascin C, thereby promoting breast cancer cell invasion; tumor metastasis and progression are associated with high integrin α11 and PDGFRβ expression in stromal cells of breast cancer [34]. In addition, single-cell RNA sequencing revealed that CAFs could be clustered into inflammatory-CAFs and myofibroblast-like CAFs in triple-negative breast cancers; inflammatory-CAFs are involved in cytotoxic T-cell dysfunction and exclusion, suggesting an important role for the CAF-related immunosuppressive environment of triple-negative breast cancers [35]. Therefore, we need to distinguish the fibroblast subpopulations in further research to better target those CAFs that secrete high levels of IL-6.

Recently, monoclonal antibodies for IL-6 or IL-6R have been investigated in clinical trials and positive results were obtained, leading to their approval by the FDA [36]. For example, the IL-6 antibody siltuximab have been approved by FDA to treat multicentric Castleman disease [37, 38]. The FDA approved the IL-6R monoclonal antibody, Tocilizumab, to treat rheumatoid arthritis [21, 22]. The results showed that tocilizumab treatment blocked the growth and radioresistance induced by CAFs in mouse models. These results suggested that combining these monoclonal antibodies with radiotherapy might be a possible approach to treat breast cancer.

Several reports showed that IL-6 and p-STAT3 levels were associated with poor clinical prognosis in several kinds of cancer [39–41]. However, few studies have investigated their association with the response of breast cancer cells to radiotherapy. In the present study, we observed that patients with high expression of IL-6 in their tumor had worse disease-free survival compared with those with low expression of IL-6 in tumor. Moreover, we found that patients with high levels of p-STAT3 had shorter disease-free survival compared with those with low levels of p-STAT3. These results indicated that in breast cancer, a poor radiotherapy response was associated with high IL-6 and p-STAT3 levels. However, whether the overall survival of radiotherapy-treated patients with breast cancer is associated with L-6 and p-STAT3 levels requires further study.

To summarize, CAF-secreted IL-6 activates STAT3 signaling, which promotes breast cancer cell growth and radioresistance. IL-6 and p-STAT3 levels correlated with a poor radiotherapy response in patients with breast cancer. CAF-induced growth and radioresistance of breast cancer cells in the TME is mediated by IL-6, thus in breast cancer, the IL-6-STAT3 signaling pathway is an important therapeutic target for radiosensitization.

## Materials and methods

### Cell lines and cell culture

The Cell Bank of the Type Culture Collection of the Chinese Academy of Sciences (Shanghai, China) provided the T47D, MCF-7, and MDA-MB-231 cells, which were grown according to standard procedures. MCF-7 and T47D cells were cultured in Dulbecco’s modified Eagle’s medium (DMEM) (Sigma-Aldrich, St. Louis, MO, USA), supplemented with 10% fetal bovine serum (FBS) (Gibco, Melbourne, Australia). MDA-MB-231 cells were cultured in Roswell Park Memorial Institute (RPMI)-1640 medium containing 10% FBS. All cells were maintained in a humidified incubator containing 5% CO2 at 37 °C.

### Fibroblast culture and isolation

We obtained fresh samples of tumors from patients with breast cancer who underwent surgery at Nanfang Hospital of the Southern Medical University. Briefly, the tumor samples were rinsed thrice using phosphate buffered saline (PBS). The samples were then cut into ~1 mm^3^ pieces followed by digestion with collagenase I, hyaluronidase, and DNase I for 2 h at 37 °C. The digested samples were filtered through a 40 mm cell strainer and the suspension was centrifuged at 1000 × g for 3 min to collect the fibroblasts. The fibroblasts were resuspended, seeded into 10 cm dishes, and cultured with DMEM containing 10% FBS. All the procedures were carried out according to the institutional guidelines of the internal review and ethics boards of Nanfang Hospital of Southern Medical University.

### Collection of conditioned medium

To collected conditioned medium from CAFs, cells were seeded into 10 cm culture dishes and incubated for 48 h. When the cells reached approximate confluence, the culture medium was collected and centrifuged at 1000 × g for 3 min at 4 °C to remove unattached cells. The conditioned medium was kept at −80 °C until use.

### Antibodies and reagents

Primary antibodies against STAT3 (#9139 T), p-STAT3 (#9145 T), FAP (#66562 S), αSMA (#19245 T), Ki67 (#9449 S), IL-6 (#12153 S), and β-actin (#4970 S) were obtained formed Cell Signaling Technology (Danvers, MA, USA). Secondary anti-rabbit IgG (#CW0103) and anti-mouse IgG (#CW0102) were purchased from CoWin Biosciences (Guangzhou, China).

IL-6–neutralizing antibodies (#MA5-23698) were obtained from Invitrogen (Carlsbad, CA, USA). Recombinant IL-6 (#PHC0063) were obtained from Gibco (Melbourne, Australia). The STAT3 inhibitor Stattic (# S7024) and the IL-6R monoclonal antibody Tocilizumab (#A2012) was purchased from Selleck (Houston, TX, USA).

### Clonogenic survival assay

Exponentially growing breast cancer cells were seeded in six-well plates in triplicate and allowed to attach for 12 h. The cells were cultured with control medium or conditioned medium from CAFs. For the combination treatment groups, the cells were exposed to IL-6 neutralizing antibodies (500 ng/ml) or the STAT3 inhibitor, Stattic, (10 μmol/ml) for 12 h following irradiation. After 2–3 weeks the colonies were fixed using 4% paraformaldehyde followed by staining with % crystal violet. After staining, we counted colonies comprising >50 cells and generated survival curves using the multitarget single-hit model.

### Immunofluorescence

Cells were seeded into 24 well plates in triplicate. After 24 h, 4% paraformaldehyde was used to fix the cells and 0.1% Triton X-100 was used to permeabilized the cells for 15 min. Following washing with PBS, the cells were incubated in the presence of primary antibodies overnight at 4 °C. Next day, the cells were washed with PBS, followed by incubation with Alexa Fluor-conjugated secondary antibodies at room temperature for 1 h. Finally, 4',6-diamidino-2-phenylindole (DAPI) counterstaining of nuclei was carried out. Immunofluorescence images were captured under a fluorescence microscope (Olympus BX51, Tokyo, Japan).

### Cytokine array

Human Cytokine Array C5 (Raybiotech, Peachtree Corners, GA, USA) was used according to the manufacturer’s instructions. Briefly, blocking buffer was incubated with the array for 30 min, followed by incubation with the samples for 2 h at room temperature. Then, the membranes were incubated with diluted biotin-conjugated antibodies, followed by reaction with horseradish peroxidase-conjugated streptavidin and exposure to X-ray film. Array Vision Evaluation 8.0 (GE Healthcare Life Science) was used to carry out the quantitative array analysis.

### ELISA

To detected IL-6 production by CAFs and NAFs, cells were seeded in a six-well plate cultured at incubated at 37 °C and 5% CO2 humidified atmosphere. As the cells grown to 80% confluence, then cultured with fresh serum-free media and the supernatant was collected at 24 h. The IL-6 ELISA Kit was purchased from Boster Biological Technology (Pleasanton, CA). The measurement according to the manufacturer’s instructions.

### Western blotting

Cells were washed with PBS then lysed in lysis buffer containing proteinase and phosphatase inhibitor (Cell Signaling Technology). The protein concentrations were detected by BCA Protein Assay Kit (Pierce Biotechnology, Rockford, IL, USA). Protein samples were subjected to SDS-PAGE followed by transfer to polyvinylidene difluoride membranes (Bio-Rad, Hercules, CA, USA). 5% nonfat dried milk was used to block the membranes for 4 h, followed by overnight incubation with the primary antibodies at 4 °C. Then, labeled secondary antibodies were incubated with the membranes at room temperature for 1 h. Finally, the immunoreactive protein bands were detected using an enhanced chemiluminescence method (Pierce Biotechnology).

### Animal study

The animal experiments were approved by the Committee on the Ethics of Animal Experiments of Southern Medical University (Guangzhou, China). MDA-MB-231 cells (1 × 10^5^) alone, or mixed with CAFs (4 × 10^5^), were injected into the right limb of the nude mice. After the tumors reached 150 mm^3^, the mice were assigned randomly into four groups and exposed to five treatments of 2 Gy radiation each time. To assess the effect of inhibiting the IL-6 receptor, an IL-6 receptor antibody, tocilizumab, (10 μg/g) was administered via intraperitoneal injection at 1 h before radiation. Tumor diameters were measured every 2 days according to the formula: (length × width^2^)/2.

### Immunohistochemical staining

Samples were fixed in formalin, embedded in paraffin, and sectioned at 4 mm thickness. The sections were deparaffinized in xylene and rehydrated in ethanol solutions. Then, the sections were incubated with 3% H_2_O_2_ for 10 min to quench endogenous peroxidase activity. Antigen retrieval in the sections was performed by microwaving for 5 min, followed by blocking using 10% goat serum for at room temperature for 1 h. Primary antibodies were then incubated with the sections overnight at 4 °C. Next day, the sections were stained using secondary antibodies, followed by incubation with an avidin-biotin peroxidase complex (GeneTex, Irvine, CA, USA). Staining extent was scored according to fraction of positive cells using the following standards: 1 (1–25%), 2 (26–50%), 3 (51–75%), and 4 (76–100%); and the staining intensity was scored as 0 (negative), 1 (weak), 2 (medium) and 3 (strong) for the staining intensity. The total score = (staining extent score) × (staining intensity score). Total score ≥8 was considered as high expression, while score <8 was rated as low expression. The tissue sections stained with p-STAT3 or Ki67 antibodies were photographed under a 200-fold microscope. Three visual fields were randomly selected to count the number of p-STAT3 and Ki67 positive cells. Finally, the positive rate was calculated. Because p-STAT3 and Ki67were mainly located in the nucleus, the number of positive-staining nuclei was counted in this experiment.

### Statistical analysis

The statistical analyses were performed using SPSS 20.0 software (IBM Corp., Armonk, NY, USA). All the data are presented as means ± standard deviation (SD) and Student’s *t*-tests or analysis of variance (ANOVA) were used to calculate the statistical significances. Kaplan–Meier survival analysis was performed to analyze disease-free survival and the statistical significance was analyzed using a log rank test. *P* < 0.05 was considered statistically significant.

## Supplementary information

Supplementary Material

## Data Availability

The corresponding author will provide the original data used to support the findings of this study upon reasonable request.
