# Supplementary material for: Cancer-associated fibroblasts induce growth and radioresistance of breast cancer cells through paracrine IL-6
Source: Cell Death Discov. 2023 Jan 13;9:6. doi: 10.1038/s41420-023-01306-3 (PMC9837084; doi:10.1038/s41420-023-01306-3)
Supplement: Supplementary file 1 — Supplementary Material [file 41420_2023_1306_MOESM1_ESM.docx]

**Supplemental Tables**

**Table S1.** **Clinical characteristics of breast cancer patients whose tumor tissues were used to CAFs and NFs isolation**

| **Patients ID** | **Age** | **Histology grade** | **Subtype** | **Tumor size(cm)** | **Axillary LN** |
| --- | --- | --- | --- | --- | --- |
| CAF1 | 32 | Ⅱ | Luminal B | 4.0 | Negative |
| CAF2 | 49 | Ⅱ | Luminal A | 2.0 | Positive |
| Case 1 | 45 | Ⅰ | Luminal A | 1.5 | Negative |
| Case 2 | 43 | Ⅱ | Luminal B | 3.5 | Negative |
| Case 3 | 54 | Ⅱ | HER2 Positive | 2.7 | Positive |
| Case 4 | 75 | Ⅲ | Luminal B | 2.4 | Negative |
| Case 5 | 62 | Ⅲ | Triple Negative | 3.0 | Positive |

**Table S2. Clinical pathologic factors of breast cancer patients**

| **Variables** | **Number** | **Percentage %** |
| --- | --- | --- |
| **Age** |  |  |
| <35 | 12 | 11.7 |
| 35-65 | 68 | 66. |
| >65 | 23 | 22.3 |
| **Tumor size** |  |  |
| ≦2cm | 26 | 25.2 |
| 2-5cm | 58 | 56.3 |
| >5cm | 19 | 18.4 |
| **Tumor subtype** |  |  |
| Luminal A | 39 | 37.8 |
| Luminal B | 36 | 35 |
| HER2 Positive | 19 | 18. |
| Triple Negative | 9 | 8.7 |
| **Histology grade** |  |  |
| Ⅰ | 14 | 16.5 |
| Ⅱ | 47 | 45.6 |
| Ⅲ | 39 | 37.9 |
| **TNM stage** |  |  |
| Ⅰ | 24 | 23.3 |
| Ⅱ | 47 | 45.6 |
| Ⅲ | 32 | 31 |
| **Lymph node metastasis** |  |  |
| negative | 31 | 30 |
| positive | 72 | 70 |
| **Margin after surgery** |  |  |
| negative | 103 | 100 |
| positive | 0 | 0 |

**Supplemental Figures**

**
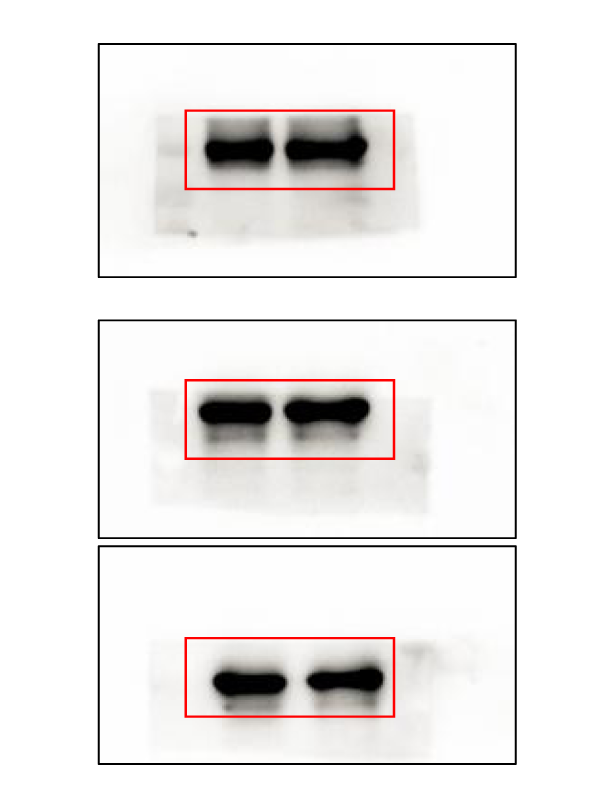
**

**Fig. S1 Original full length western blots in Figure 1B.**

**
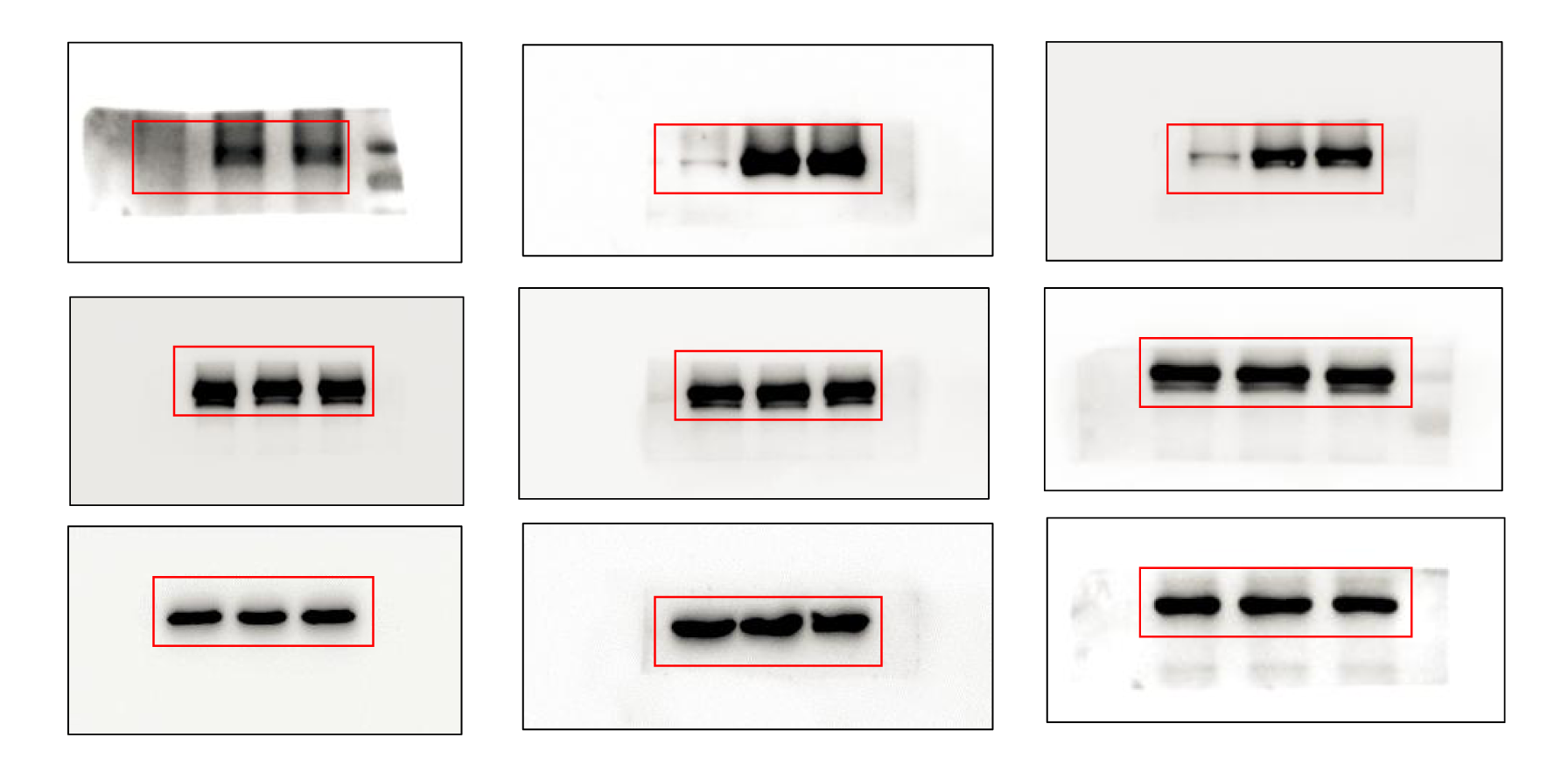
**

**Fig. S2 Original full length western blots in Figure 3C.**

**
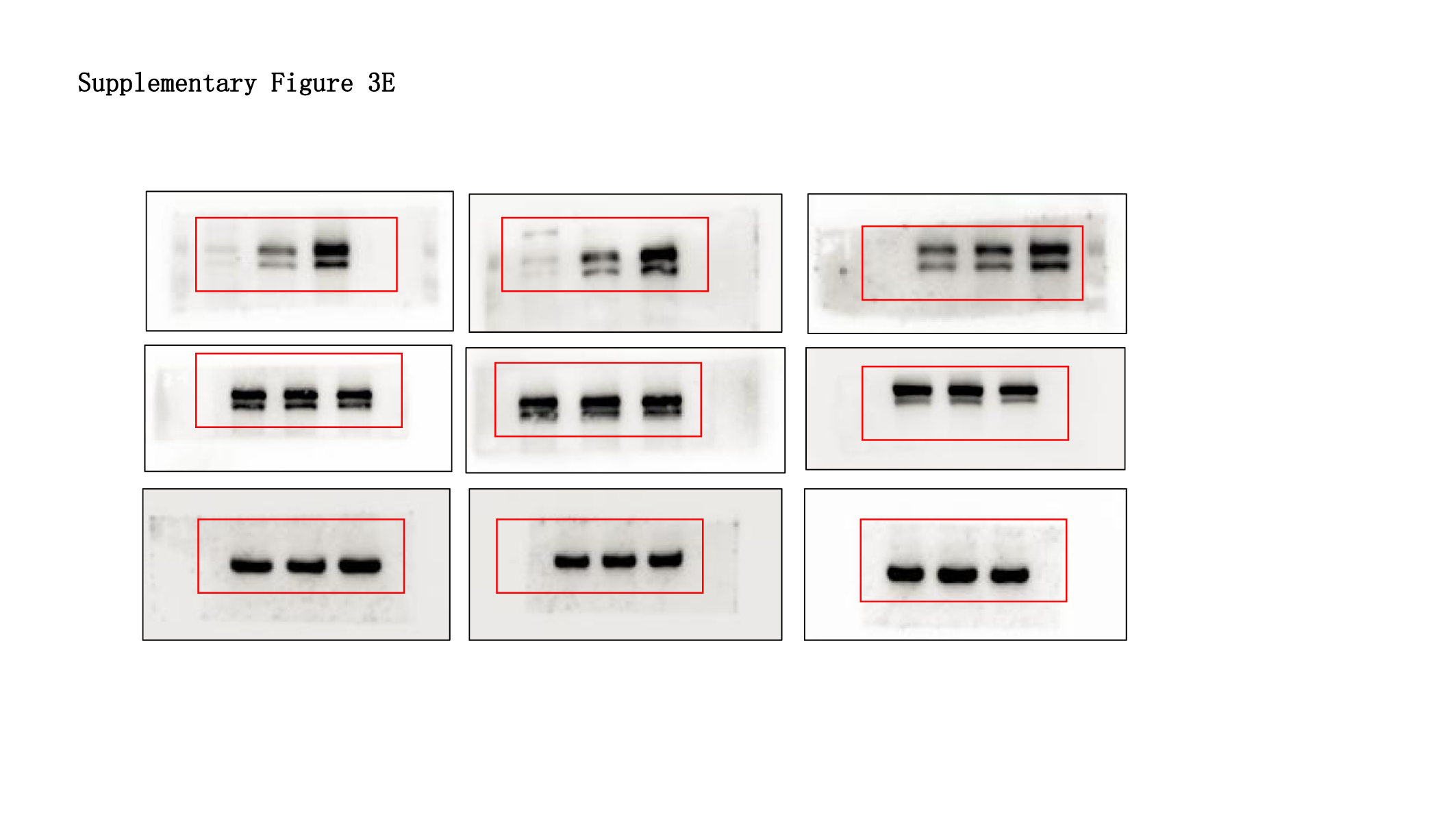
**

**Fig. S3 Original full length western blots in Figure 3E.**

**
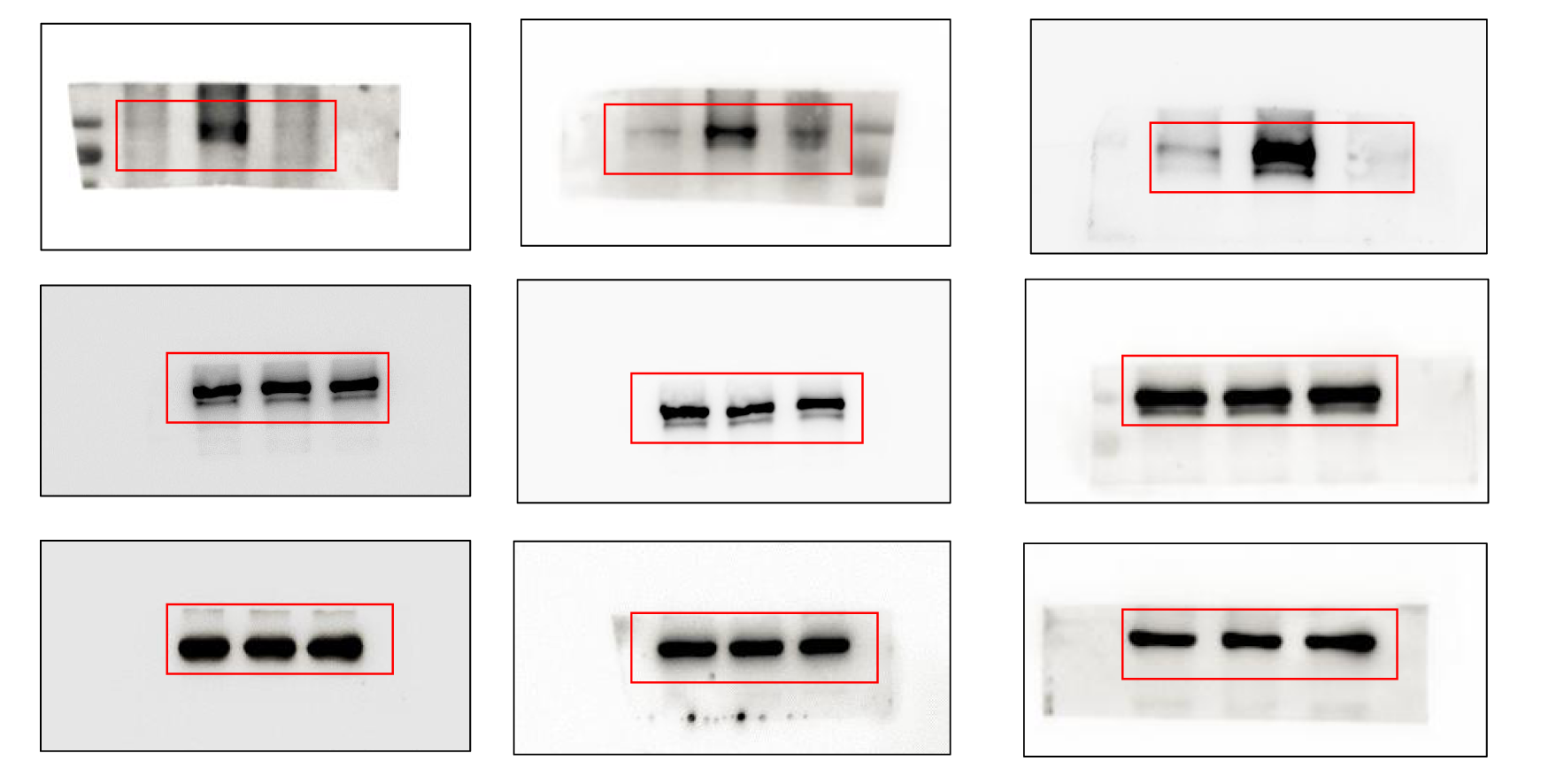
**

**Fig. S4 Original full length western blots in Figure 4A.**

**
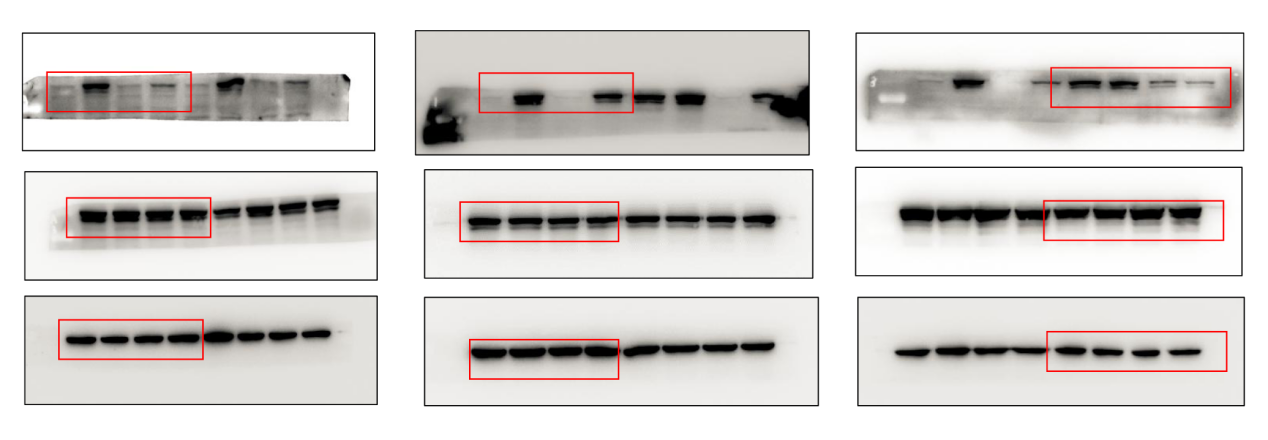
**

**Fig. S5 Original full length western blots in Figure 5A.**
